# Supplementary material for: Are carbon emissions trading and green financial instruments synergistic? -Comprehensive quantitative research based on content analysis
Source: PLoS One. 2024 Mar 7;19(3):e0298601. doi: 10.1371/journal.pone.0298601 (PMC10919601; doi:10.1371/journal.pone.0298601)
Supplement: S1 Data — (ZIP) [file pone.0298601.s001.zip › Data/地级市Z_F工作报告.zip!/╔╜╢1⁄2/┴─│╟╩╨/└┤╫╘╣1⁄2╓┌║┼í╛┬φ┐╦╩2╛▌═°í┐_2007.docx]

各位代表：

　　现在，我代表市人民政府向大会作政府工作报告，请予审议，并请市政协委员和其他列席会议的同志提出意见。

一、2006年工作回顾

　　2006年，是“十一五”开局之年。全市人民在省委、省政府和市委的坚强领导下，高举邓小平理论和“三个代表”重要思想伟大旗帜，认真贯彻党的十六大和十六届三中、四中、五中、六中全会精神，全面落实科学发展观，解放思想，干事创业，开拓进取，扎实工作，改革开放和现代化建设取得新的成就，实现了“十一五”良好开局。

　　——全市生产总值达到839亿元，比上年增长17.3%，人均生产总值达到1920美元。

　　——财政总收入76.1亿元，其中地方财政收入33.6亿元，增长30.4%。

　　——社会消费品零售总额271.8亿元，增长16.4%。居民消费价格总水平上涨1.4%。

　　——新增城镇就业再就业9.4万人，转移农村劳动力10.5万人。

　　——城镇居民人均可支配收入10474元，农民人均纯收入3948元，分别增长16.1%和14.5%。年末城乡居民本外币储蓄余额403.9亿元，比年初增加43.8亿元。

　　——文明聊城、平安聊城、诚信聊城、和谐聊城建设进一步加强，全市政治安定、社会稳定，人民群众安居乐业。

　　一年来，我们主要做了以下几方面的工作：

　　（一）认真贯彻国家宏观调控政策，经济增长质量明显提高。全面落实中发14号文件精神，严把土地、信贷闸门和市场准入门槛，全面清理新开工项目，着力优化投资结构，努力消除经济发展中的不稳定、不健康因素。规模以上固定资产投资完成350.9亿元，增长19.3%，其中100个重点项目完成投资139.6亿元，较好实现了宏观调控目标。投资结构进一步优化，重点投资领域得到加强，农业和服务业领域的投资分别增长159%、13.2%。信发华宇氧化铝粉、时风农业装备公司、鲁西化工40万吨尿素扩建、希杰核苷酸二期扩建、鲁能热电2×300MW机组、东阿阿胶7000吨阿胶浆扩建等一批重点项目建成投产。

　　（二）高度重视“三农”工作，社会主义新农村建设开局良好。认真落实各项支农惠农政策，全部取消农业税。实行大型农机具、良种、粮食直补和农资补贴，仅此农民人均受益74.1元。争取国家土地开发整理资金2.1亿元。加大农村基础设施投入，各级财政资金共投入2.9亿元。改造农村公路1282.5公里，自来水入户普及率达到66%。大力提高农业综合生产能力，粮食总产423.9万吨，增长5.7%；蔬菜总产826.6万吨，增长8.2%。积极推进农业产业化，规模以上农业龙头企业达到340家，增加73家。实施了“万村千乡”市场工程和“百千万”帮促工程。科技特派员工程向纵深发展，培育示范基地200多个，带动30多万农户，增加效益6500多万元。

　　（三）积极推进结构调整，经济整体素质进一步增强。认真实施工业带动战略，大力发展服务业，三次产业结构调整为16.5∶58.6∶24.9，二三产业比重提高2.18个百分点。认真实施“5513”工程，大力推进“三个一批”，规模以上工业企业比上年增加345户，完成增加值393.4亿元、利税145.6亿元、利润93.6亿元，分别增长28%、53%和53％；新增中国名牌4个、山东省名牌28个、地理标志产品1个。旅游、商贸物流、交通运输、文化、金融等产业发展较快。旅游总收入和旅游人次分别增长16％和12.8％，东昌湖风景区被评为国家4A级景区。

　　（四）加强自主创新、节能降耗和环境保护，经济增长方式进一步转变。制定实施了《聊城市中长期科技发展规划纲要（2006－2020）》，全面推进创新型城市建设。争取国家“863”相关项目10项。取得科技成果186项、发明专利484项，分别增长47.6%和91.3%。建成博士后科研工作站4家、省级以上技术中心16家。高新技术产业产值达到285.8亿元，占规模以上工业总产值比重提高2.1个百分点。大力推进资源节约型、环境友好型社会和生态市建设，突出抓好50户重点企业节能和技术改造。万元生产总值能耗、规模以上工业万元增加值能耗基本完成控制目标。主要污染物化学需氧量、二氧化硫排放量分别较上年减少4.5%和2.5%。

　　（五）大力推进改革开放，经济发展活力明显增强。深化农村综合改革、国有企业改革和国有资产管理体制改革。顺利完成国有粮食购销企业改革。加快财税体制改革，市级全面推行了国库集中收付制度。积极推进地方金融机构改革，完善了农村信用社法人治理结构。民营经济保持快速健康发展势头，主营业务收入、利税分别增长47%和60.5%。稳步推进事业单位改革，乡镇卫生院上划县级管理工作基本完成。继续下大气力抓好招商引资和对外开放，实际到位招商资金136.7亿元，按可比口径增长12.9%；实际利用外商直接投资6945万美元。积极扩大进出口，进出口总额9.95亿美元，增长56.1%，其中出口6.1亿美元，增长32.8%。机电产品和高新技术产品出口比重分别提高4.4和0.15个百分点。实施“走出去”战略，海外承包工程有了良好开端。

　　（六）精心打造城市品牌，城市建设步伐明显加快。坚定不移地打造“江北水城”这一品牌，提升城市综合竞争力。全市城市建设投资完成21亿元，其中聊城城区6亿元。精心组织实施聊城城区建设十大工程，完成了建设路、花园路、站前街等道路的拓宽改造和新水河、青年渠截污治污工程，实施了东昌湖风景区完善、运河四期开发、人民公园改造等工程。城区新增管道燃气居民用户8500户、供热面积150万平方米、绿化面积390万平方米。创建国家环保模范城和国家卫生城取得阶段性成果。全市城市化水平达到36.9%，增加2.9个百分点。

　　（七）坚持统筹协调发展，社会事业全面进步。在完善“以县为主”的义务教育管理体制、调整学校布局的同时，全市投入2160万元，用于农村中小学课桌凳更新和“两免一补”，投入1.15亿元，新建高标准中小学校舍17.9万平方米；初中升学率达到79.7%，提高21.7个百分点；高考升学率53.7%，本科上线18436人，增加2386人。加快公共卫生“两个体系”建设，投入1000万元，完成了10所乡镇中心卫生院建设改造，新型农村合作医疗投入1216.5万元，参合农民达202.8万人。加强文化大市建设，成功举办了“‘五一’文化旅游节”、“‘十一’水城之秋观光周”和首届“江北水城合唱节”。聊城杂技列入国家第一批非物质文化遗产。承办了乒乓球超级联赛等国内重大赛事，在省第21届运动会上取得良好成绩。圆满完成人口控制计划，人口自然增长率控制在4.43‰。争创全国双拥模范城“二连冠”工作取得阶段性成果。广电、出版、外侨、对台、民兵、国防动员、民族宗教、妇女儿童、残疾、档案、史志、人防、防震减灾等工作都取得新的进展。

　　（八）着力解决群众实际困难，积极构建和谐社会。切实抓好就业再就业工程，城镇登记失业率控制在3.15%。社会保险覆盖范围进一步扩大，“两个确保”得到较好落实，城市低保实现应保尽保，提高了城市最低工资标准，实施了城市医疗救助制度。稳步推进农村“五保”财政供养，建设中心敬老院18处。切实关心弱势群体，解决建设领域拖欠农民工工资980万元，建成经济适用房10.6万平方米。强化区域经济协调发展，3个欠发达县和马西地区发展步伐加快。认真落实安全生产“一岗双责”，安全事故起数、死亡人数分别下降6.2%和9.4%。加强社会治安综合治理，深入推进“平安聊城”建设，实现了社会和谐稳定。

　　（九）大力推进政府管理创新，切实提高依法行政水平。积极实施依法行政“四五”规划，相对集中行政处罚权和行政许可权。继续完善行政审批制度改革，按时办结率和服务满意率分别达到100%和99%。大力推进政务公开，加强政务督查，完善听证制度、专家咨询制度，努力畅通与人民群众的全天候联系渠道。初步建立了突发公共事件应急管理体系。认真组织实施反商业贿赂工作，查处了一批违法违纪案件。在建设阳光政府、服务政府、责任政府、法治政府方面向前迈出了坚实的步伐。

　　各位代表，在过去的一年里，我们之所以能够取得这样优异的成绩，是党中央、国务院和省委、省政府正确领导和亲切关怀的结果，是市委科学决策、正确领导的结果，是人大、政协和社会各界监督支持的结果，是全市人民团结一心、干事创业、辛勤工作的结果。在此，我代表市人民政府，向全市工人、农民、知识分子、各级干部，向各民主党派、工商联、无党派人士和社会团体，向驻聊人民解放军、武警部队官兵、公安干警和中央、省驻聊单位，向所有关心和支持聊城发展，在我市投资创业的海内外朋友，表示衷心的感谢和崇高的敬意！

　　各位代表，在充分肯定成绩的同时，我们也清醒地看到，我市经济社会发展还存在不少问题和困难，政府工作也存在不少缺点和不足，主要是：经济总量仍然较小，结构性矛盾较为突出，先进制造业、高新技术产业比重较低；投资规模仍然偏小，投资结构不够合理；经济增长方式较为粗放，自主创新能力不强，资源、环境压力较大；对外开放度仍然不高，融资能力不强；体制机制性障碍仍然存在，发展环境有待进一步改善；统筹发展能力较弱，区域发展不够平衡，农民持续增收的长效机制尚未形成，就业压力较大，社会保障水平较低；有些部门依法行政的观念不强，工作效率不高。对此，我们一定要高度重视，采取切实措施，认真加以解决。

二、2007年政府工作的总体要求

　　2007年是深入贯彻落实科学发展观、扎实推进社会主义和谐社会建设的重要一年，党的十七大和省第九次党代会、市十一次党代会将在年内召开。做好今年的政府工作，保持经济社会发展的良好势头，意义十分重大。

　　综合分析今年的宏观形势，我们既面临前所未有的重大发展机遇，又面临严峻挑战，总的看，机遇大于挑战。从机遇方面来看，一是世界经济继续保持较快增长，特别是高新技术产业和现代服务业加速向新兴市场和发展中国家转移，为我市承接国际产业转移、加速工业化进程带来了新的机遇；二是国家继续加强和改善宏观调控，为我市调整优化经济结构、转变经济增长方式、提高经济质量和效益提供了难得的机遇；三是中央、省统筹城乡和区域经济发展的力度加大，对农业地区和相对欠发达地区给予更多的政策支持，为我们争取更多的政策、资金、项目提供了难得的机遇；四是我市经济已开始进入发展的快车道，信发、时风、阿胶、鲁化等骨干企业进一步做大做强，祥光、奥博特、冠洲、冠星等一批新的大项目迅速崛起，加之“江北水城”品牌的成功打造，为我市加快推进工业化、城市化奠定了坚实基础；五是全市广大干部群众团结一心、人心思上，想发展、盼发展，想干事、会干事，聊城人比历史上任何时候都更加自信和豪迈，这是聊城站在新起点、实现新发展的最大机遇和最强大的动力。另外，中央提出发展天津滨海新区，山东提出打造省会城市群经济圈，北京承办2008年奥运会，这一切为我市加快接轨济南、融入省会城市群经济圈和环渤海经济圈，分享区域经济共同发展带来的繁荣，创造了极佳的发展机遇。

　　从挑战方面来看，一是国际贸易保护主义抬头，国际初级产品价格在高位运行，加入世贸组织过渡期结束，国家履行服务业各项承诺，降低进口关税，调整部分商品出口退税率，这对我们应对国内外市场竞争是一个严峻的挑战；二是土地、资金供求趋紧，节能环保等要求提高，对我们转变增长方式、提高发展质量提出了更高的要求；三是改革发展进入新阶段，如何及时消除不和谐因素，实现经济社会持续快速协调健康发展，对我们提高执政能力、驾驭能力带来新的考验；四是各地都在你追我赶、竞相发展，给我们带来的竞争压力越来越大。

　　面对新机遇、新挑战，我们一定要倍加珍惜改革开放以来特别是近十年来的好经验、好做法，倍加珍惜来之不易的好局面、好成果，倍加珍惜人心思上、人心思干的好风气、好状态，动员全市573万人民，紧紧围绕建设强市名城的宏伟目标，万众一心，众志成城，扎实苦干，奋力拼搏，不断开创各项工作的新局面，努力把聊城建设得更加生机勃勃、更加稳定和谐、更加繁荣昌盛。

　　根据市委的部署，政府工作的总体要求是：坚持以邓小平理论和“三个代表”重要思想为指导，深入学习贯彻党的十六大和十六届三中、四中、五中、六中全会精神，全面落实科学发展观，深入贯彻国家宏观调控政策，着力推进社会主义新农村建设，着力加快工业化和城市化步伐，着力调整经济结构和转变增长方式，着力加强资源节约和环境保护，着力推进改革开放和自主创新，着力促进社会发展和解决民生问题，努力实现经济社会又好又快发展，加快构建社会主义和谐社会，为党的十七大和省、市党代会召开创造良好的经济环境、政治环境和社会环境。2007年经济社会发展的主要预期目标为：全市生产总值增长15%；社会消费品零售总额增长15%；地方财政收入增长20%，突破40亿元；高新技术产值占规模以上工业总产值的比重达到21%；全社会固定资产投资增长20%；外商直接投资增长45%；外贸出口增长20%；万元生产总值能耗下降4.8%，二氧化硫排放减少3.5%，化学需氧量排放降低5%；城镇登记失业率控制在3.5%以内；城镇居民人均可支配收入增长10%，农民人均纯收入增长8%；居民消费价格总水平涨幅控制在3%以内；人口自然增长率控制在7‰以内。

　　在工作指导上，要切实把握好以下几个方面：

　　——必须牢牢抓住落实科学发展观和宏观调控这一机遇不放松，做到又好又快、好中求快，努力实现科学发展、跨越发展。

　　——必须牢牢抓住“三农”问题不放松，大力发展现代农业，努力增加农民收入，扎实推进社会主义新农村建设，夯实经济社会又好又快发展的基础。

　　——必须牢牢抓住增加优质高效投入不放松，特别是抓大项目、上好项目，壮大整体经济实力，努力增强经济发展后劲。

　　——必须牢牢抓住工业带动战略和“一把手”工程不放松，努力建设“三个一批”，大力发展产业集群，加速推进工业化进程，努力夺取未来发展主动权。

　　——必须牢牢抓住民营经济和改革开放不放松，着力推进体制机制创新，努力增强经济社会发展的动力和活力。

　　——必须牢牢抓住自主创新、节能降耗、环境保护、集约用地不放松，创新发展模式，转变增长方式，努力建设创新型城市和节约型社会。

　　——必须牢牢抓住统筹兼顾不放松，大力推进城乡之间、经济社会之间、区域之间协调发展，全面促进社会进步。

　　——必须牢牢抓住以人为本、关注民生不放松，把实现好、维护好、发展好最广大人民的根本利益作为工作的出发点和落脚点，努力构建和谐社会。

三、2007年经济社会发展的重点

　　（一）积极发展现代农业，扎实推进社会主义新农村建设

　　聊城是农业大市，社会主义新农村建设是长期的战略任务。要把解决“三农”问题始终作为全部工作的重中之重，把提高农业综合生产能力和增加农民收入始终作为“三农”工作的重中之重，全面落实工业反哺农业、城市支持农村和多予少取放活的方针，促进农业不断增效、农村加快发展、农民持续增收。

　　大力发展现代农业。用现代产业体系发展农业，在严格保护耕地、稳定粮食供求关系的基础上，进一步调整优化农业产业结构，大力发展蔬菜、果品、食用菌、花卉等高效农作物；大力发展林牧渔业，尤其是畜牧业占农业总产值的比重要有明显提高；大力发展农村二、三产业。用现代物质手段装备农业，用现代科学技术改造农业，积极推进农技推广体系改革，继续大力实施科技特派员工程，加大农业新技术、新品种、新装备的推广力度，认真实施沃土工程，大力发展生态农业，努力提高农产品质量效益和市场竞争力。用现代经营方式提升农业，通过贴息补助、税收优惠等政策，支持龙头企业发展，培育农民专业合作组织，实施“金农”工程，加快建设一批设施先进、功能完善、交易规范的农产品批发市场，完善农产品质量安全标准体系和监管体系，新创国家级农业标准化示范区2个，新增“三品”认证26个。

　　进一步加大对“三农”的投入。推进新农村建设，加大投入是关键、是保证。今年各级财政支农资金的增量要继续高于上年，固定资产投资用于农村的增量继续高于上年，土地出让收入用于农村建设的增量继续高于上年，全市财政安排“三农”投入8.5亿元，比上年增长20%。实施“百千万”帮促工程，加大支农资金整合力度，提高使用效益。今年重点做好十件实事：一是新建改造农村公路500公里，改造农村危桥148座，新建农村客运站25个；二是加快位山、陶城铺、郭口、彭楼等灌区的续建配套和节水改造，新打更新机井5000眼，新增自来水用户10万家；三是加快实施农村广播电视村村通工程，新增农村有线电视用户3万家；四是加快发展农村清洁能源，新增沼气用户3万家；五是全面建立新型农村合作医疗制度，参合农民增加到370万人，参合率达到85％以上；六是从今年起全部免除农村义务教育阶段学生学杂费，继续对农村贫困家庭学生免费提供课本并补助寄宿生生活费；七是全面建立和完善农村最低生活保障制度，保障标准每人每年不低于700元；八是新建改建19处农村中心敬老院，集中供养率力争达到70％；九是研究制定符合聊城实际的新农村建设总体规划，完成所有乡镇和60%以上中心村总体规划，抓好一批新农村建设试点；十是大力开展农村环境综合整治，改善村容村貌，突出省际间村镇改造与整治，绿化美化边界环境。

　　深化农村综合改革。扎实推进农村义务教育、乡镇机构和县乡财政体制改革，严格落实五年内乡镇机构不增加编制的规定。全面清理核实乡村债务，积极探索化解债务的措施和办法。落实对村级的转移支付，继续严格控制农村“一事一议”，推进农田灌溉用水计量收费，坚决防止农民负担反弹。加快培育农村金融市场，发挥农行、农发行在农村金融中的骨干作用，深化农村信用社改革，引导邮政储蓄等资金返还农村，扩大农户小额贷款规模，抓好临清市农业政策性保险试点。加快建立农村公路和自来水等基础设施管理体制。努力加快马西地区、黄河故道、沉沙池区、金堤河滞洪区等区域群众的脱贫步伐。

　　大力培养社会主义新型农民。落实高校毕业生到农村基层服务计划和一村一名大学生工程。充分利用各种教育资源，抓好“阳光培训”、绿色证书培训、新型农民科技创业培训，突出抓好“185”工程，集中培训农业技能辅导员1000人，发展科技带头人8000人，带动农民50万人。积极开展和谐家庭、和谐村组、和谐村镇创建活动，倡导科学健康、文明向上的生活方式。加快县级文化馆、图书馆和乡镇文化站、村文化室等农村公共文化设施建设，鼓励农民兴办文化产业。深入开展“三下乡”活动，丰富农村精神文化生活。多种形式对农民进行党的政策、移风易俗和社会主义荣辱观宣传教育，普及科学、法律知识，搞好乡规民约建设。

　　（二）坚定不移地贯彻国家宏观调控政策，努力扩大优质高效投入

　　坚持把贯彻落实国家宏观调控政策作为又好又快发展的重大机遇，努力消除经济运行中不稳定、不健康的因素，积极扩大优质高效投入，努力增强经济社会发展后劲。

　　认真贯彻落实宏观调控政策。坚定不移、不折不扣地贯彻执行中央宏观调控政策，严把土地、信贷两个闸门和市场准入门槛，强化对投资项目的源头管理，从严控制新增建设用地和农用地转用总量。同时，坚持有保有压、区别对待，把严格把关与优质服务有机结合起来，全面加强项目审批、土地供应、资金筹措等各项配套服务工作，确保重点项目及时开工建设、早日投产达效。

　　全力抓好新形势下的招商引资。没有招商引资的大突破，就没有聊城经济的大发展。要进一步统一思想，加大措施，尽快实现招商引资的新突破。要巩固日韩港台、长三角、珠三角招商阵地，开拓对京津地区和欧美地区的招商。要发挥企业招商主体的作用，力争在重点产业招商、大项目招商、产权招商方面取得更大成效。要进一步强化招商队伍建设，改进招商方式，提高招商实效，尤其要更多地采取登门招商、委托招商、网上招商、以商招商等多种有效方式，实现企业、产业、园区招商的新突破。继续举全市之力支持和加快聊城经济开发区改革、建设、发展，使之成为实现又好又快发展的最优平台和最快增长点。

　　着力优化投资结构。在努力保持适度投资力度的同时，采取切实措施，引导有限投入着重投向符合国家产业政策、节约能源资源的项目，投向科技含量高、附加值高、市场前景广阔的项目，投向有利于发展先进制造业、强化基础设施和社会事业的项目，投向有利于增加地方财政收入、扩大社会就业和增加城乡居民收入的项目。大力抓好100个重点项目建设，筛选充实一批高科技项目进入重点项目目录。大力抓好祥光阴极铜、信发铝深加工、奥博特铜深加工、泉林林浆纸一体化、鲁化工业园等重点项目建设，确保早日建成投产。搞好邯济铁路复线、德商高速、聊泰高速、聊济一级公路、莘县盐化工等项目的论证、规划和立项，争取早日开工建设。全力做好电网输变电工程建设，争取国电聊城电厂二期、鲁能热电扩建工程项目列入国家电力建设规划。

　　（三）大力发展二、三产业，努力促进经济结构优化升级

　　加快发展二、三产业是从根本上解决“三农”问题、建设强市名城、构建和谐聊城的必由之路。要牢固树立“工业立市、工业强市”理念，坚定不移地实施工业带动战略，按照“三个一批”的要求，以“5513”工程为抓手，全力建设百亿产业，发展产业集群，努力走出一条具有聊城特色的新型工业化道路。一是突出抓好重点企业。对“5513”工程核心企业在资金、土地、重组等方面给予强力支持。着眼未来，突出抓好重点企业引进战略合作伙伴，积极参与国际分工与竞争，加强企业核心竞争力建设，夯实技术、质量、管理、品牌基石，在全国同行业进三强、争第一，做百年企业。力争今年销售收入过200亿元的企业达到2户、过50亿元的4户，利税过亿元的25户、过千万元的180户，8家企业进入全省百强。东昌府区、东阿、莘县要尽快在培植骨干企业上实现突破。二是大力发展产业集群。紧紧围绕电力及有色金属、汽车及农业装备、林浆纸业、医药、食品、化工、纺织服装等七大产业链条，以重点企业为依托，搞好中小企业配套，促进产业集聚。加大用高新技术和先进适用技术改造传统产业力度，瞄准国际国内顶尖产品进行改造，抢占行业发展制高点。搞好工业园区产业空间布局和资源整合，发挥优势，突出特色，错位发展。三是大力实施品牌战略。充实完善《名牌产品培育发展规划》，支持企业采用国际标准，鼓励优势企业参与国家乃至国际标准的制定。“十一五”期间，重点培育20个技术含量高、品牌影响力强的产品，建设20家创新能力达到国内一流的企业技术中心。四是积极拓宽融资渠道。做好企业股份改造、产权出让、票据融资、利用外资、债券市场融资等工作，力争有2家企业上市。努力优化金融生态环境，积极搭建银企合作平台。充分利用市场机制，尽快实现信用担保体系发展的突破。努力改善企业融资担保结构，防范化解关联风险。

　　把服务业摆在经济社会发展更加突出的位置，作为优化结构、推进城市化、安置就业、增加财政收入的战略重点来抓，切实加大投入，创新体制机制，优化发展环境，全力推动上层次、上水平。一是做大做强旅游业。继续突出“江北水城”和国家历史文化名城的特色，着力挖掘文化内涵，尽快把聊城培育成全国旅游热点城市。打造“夜游水城”品牌，建设特色旅游街区。加快重大旅游项目建设，继续争创4A级、3A级国家风景名胜区。会同济南、泰安、曲阜共同打造“山泉水圣”旅游圈。进一步整合天沐温泉等全市旅游资源，组建几条精品旅游线。力争开通“北京—聊城”汽运旅游专线及铁路客运始发列车，吸引更多国内外游客。二是加快商贸物流业发展。加快制定聊城市城区商业网点发展规划和物流基地发展规划。大力发展第三方物流，着力抓好市中心商业区和香江市场、开发区物流配送中心及城区北部物流基地的培育和发展，着力促进烟店轴承市场上层次、上水平。争取北方铁路有限公司落户聊城。适时举办农机装备、轴承、阿胶等博览会，打造水城会展品牌。三是大力发展文化产业。完善文化产业发展政策，深化文化体制改革，更好地把文化与城建、经贸、体育、旅游结合起来，培育具有自我发展能力的大型文化企业集团，形成知名文化产业品牌。挖掘传统文化产品，发展成为现代文化产业。整合演出资源，打造精品演出剧目。继续搞好“江北水城”文化旅游节、合唱节等节庆活动，支持各县（市、区）开展各具特色的文化节庆活动。四是推进各项服务业全面发展。特别是大力发展金融保险、房地产、交通运输、餐饮休闲、信息服务、中介等服务业。

　　（四）大力发展民营经济，着力推进改革开放

　　民营经济是最具活力、最有增长潜力的市场主体，在调整经济结构、促进经济增长、扩大就业和活跃市场等方面发挥着非常重大的作用。民营活，则全局活；民营兴，则全市兴。要认真落实《中小企业促进法》等一系列政策措施，出台我市实施细则和配套文件。把民营经济作为打造产业集群的主力军，围绕百亿产业发展民营经济。选择100家成长型中小企业，给予重点扶持服务。大力抓好融资担保、信用评价、技术服务、人才培训、法律服务、行业协会等民营企业服务体系建设，2007年新增担保公司18家，实施银河培训、蓝色证书培训、“百千万”培训工程，每年培训100名民营企业家、1000名中小企业经营者、10000名职工，建立轴承、密度板、纺织、农副产品加工等行业协会。充分发挥高新技术产业园、高新技术创业服务中心和9家民营经济园区等中小企业创业基地的作用，加快中小企业的培育。大力推进中小企业在深圳中小板上市，使更多的民营企业做大做强。在全社会倡导创业之风，鼓励自主创业，鼓励发展草根经济，努力形成既有参天大树、又有茂密森林、更有茵茵绿草的良好经济生态环境。

　　改革是经济社会发展的重要动力，要靠改革创新体制机制，靠改革解决突出矛盾和问题，靠改革开创各项工作新局面。一是加快国企改革。研究提出我市国有经济布局调整指导意见，加快推进国有资产结构调整和优化重组，适时建立国有资产经营预算制度。完成东阿阿胶股权分置改革，加快聊建集团、鲁化集团、中通集团、鑫亚公司、水城集团、水务集团的改革。探索建立困难企业重组脱困机制，努力做好企业破产处置和职工安置工作，切实维护好职工合法权益和社会稳定。二是加快推进财税体制改革。完善财政奖励补助政策和县乡财政管理体制，着力缓解县乡财政困难。加快推进部门预算、国库集中支付等财政改革，搞好城镇土地使用税、车船税等税制改革，规范国有资本收益上缴和使用。强化税收执法，推进综合治税，提高税收征管质量和效益。三是推进行政事业单位改革。落实《公务员法》，实行实名制管理，严格控制行政事业单位编制，建立完善公务员管理信息系统。加大经营开发服务类事业单位转企改制力度，全面推进社会公益类事业单位用人制度和分配制度改革。四是完善现代市场体系。进一步规范土地出让、出租、抵押等交易活动，加强地价监测，促进土地市场平稳发展。强化对垄断行业价格成本的监审。加快社会信用体系建设，完善市场监管和质量监督机制。

　　开放是我国的基本国策之一，更是我市实现率先崛起的重要法宝。要靠开放，引进更多的资金、技术、人才；靠开放，不断拓宽发展的视野，不断更新发展的理念，不断创造崭新的业绩。今年是我国加入世贸组织过渡期结束后的第一年，要适应新的形势，进一步提升对外开放的层次和水平。一是努力扩大利用外资。积极探索利用外资新途径，大力推进外资并购、外资企业增资扩股和境外上市，努力争取一批利用外资大项目落户。积极探索引入BOT、BT等利用外资模式，引导外资投向高新技术产业、高端制造业、现代服务业、现代农业、基础设施和生态建设。积极利用国际金融组织和政府贷款。完善外资项目协调推进机制，重点抓好泉林纸业复合软包装二期、希杰三期赖氨酸、华润三期增资等项目建设。二是加快转变对外贸易增长方式。落实出口退税政策，调整出口产品结构，实施出口品牌战略，扩大机电产品、纺织品和农产品出口，着力抓好“5513”工程企业产品出口。组织企业积极参加国内外展洽活动，巩固日韩、港台市场，扩大欧美市场，大力开拓非洲、拉美市场。引导企业运用人民币升值后带来的价格优势，扩大紧缺资源和先进技术、关键设备进口。建立完善反倾销应诉支持机制和出口产品预警机制，提高应对国际贸易摩擦的能力。加快聊城市保税物流中心和陆路口岸建设，建立符合国际惯例的配套物流服务体系。三是大力发展区域合作。研究制定融入省会城市群经济圈的战略规划，加快与济南接轨的步伐，今年要取得实质性进展。抓住我市加入环渤海经济联合市长联席会和北京承办2008年奥运会的机遇，主动承接环渤海发达地区的辐射和产业转移。四是积极实施“走出去”战略。支持各类有条件的企业扩大境外投资，开发铝土矿、铜精矿等紧缺资源，建立资源基地，确保资源可持续供应。继续抓好对外承包工程和外派劳务工作，确保安全，创出品牌，提高效益。大力做好外事工作，加强与友好城市的往来与经贸合作，不断谱写对外合作的新篇章。

　　（五）切实提高自主创新能力，努力建设创新型城市

　　创新是一个民族进步的灵魂，更是一个地区保持生机与活力的不竭动力和源泉。要大力实施科教兴聊和人才强市战略，全面实施《聊城市中长期科技发展规划纲要（2006—2020）》，坚持开放发展科技，致力于形成良好的创新文化和创新环境，努力走出一条符合聊城实际的自主创新之路。

　　加快推进科技自主创新。一是大力发展高新技术产业。以电子信息、新材料和生物工程为重点，培植高新技术产业群。集中力量抓好混合动力客车、电动客车、绿色环保橡胶助剂等10个“863”相关项目及其他高新技术产业化项目，力争今年高新技术企业达到80家以上。逐步增加市县两级财政研发投入，设立市高新技术风险投资基金，鼓励企业加大科技开发投入。高新技术产业产值比重力争增加2个百分点。二是努力打造科技发展平台。加快高新技术产业园发展，力争市高新技术创业服务中心成为国家级高创中心。抓紧建设院士工作站。三是着力培育自主创新主体。建立以企业为主体、产学研结合的技术创新体系，鼓励支持企业与高校院所共建技术研究中心、重点实验室、博士后工作站等研发机构，大力开发具有自主知识产权的关键技术和核心技术。

　　大力实施人才强市战略。加强对企业经营管理人才、高级专业技术人才的培训，培养一大批优秀企业家和学科带头人。全面落实《聊城市引进高层次人才暂行办法》，吸引高层次人才以柔性流动等形式支持聊城发展。深化人才制度改革，建立鼓励优秀人才脱颖而出的有效机制，努力营造人尽其才、才尽其用的良好社会环境。

　　积极推进国民经济和社会信息化。落实《聊城市国民经济和社会信息化“十一五”发展规划》，全力实施“1142”信息化发展战略。统筹规划建设信息基础设施，建设“数字聊城”，实现资源共享，确保信息安全。扎实推进电子政务建设，自7月1日起实行市级电子公文传输，加快全社会信息化步伐。

　　（六）加强资源节约和环境保护，努力实现经济社会可持续发展

　　资源节约和环境保护是经济行为、社会行为，也是道德行为。要大力倡导健康、节约、环保的生产、消费模式和社会风气，积极建设资源节约型和环境友好型社会，真正造福当代、惠及后人。

　　突出抓好节能降耗。严格落实《聊城市资源节约标准》，以节能节水节地节材和资源综合利用为重点，突出抓好电力、有色金属、化工、造纸等高耗能行业和50户重点企业的节能工作。抓好清洁生产，发展循环经济，大力实施“2320”工程。大力推广节水型农业，新增节水灌溉3.6万亩，推行城市分类供水、中水回用和一水多用，建立完善用水累进加价制度。发展节能省地型住宅,加大推广新型墙材和禁用实心黏土砖工作力度，严格执行民用建筑节能65%、公用建筑节能50%的设计标准。加强土地管理，提高土地集约利用水平，开发区每亩投资应在150万元以上，各县（市、区）工业园区应在100万元以上。

　　着力加强环境保护。坚持预防为主、综合治理，积极推进生态市、生态县、生态示范区建设。加强环保执法，严格执行环境影响评价和“三同时”制度，全面实行排污总量控制和排污许可证制度，定期公布环境质量、环境管理和污染治理信息。加强对城乡生活饮用水水质和城市空气质量的监控检测。以“四河一湖”碧水行动为重点，推动重点流域污染防治，全面完成聊城市第二污水处理厂等5座污水处理厂建设。强化大气污染治理，全面完成28家电厂脱硫工程建设，加快国电中华电厂脱硫工程建设。大力实施农业“两减三保”计划，抓好农业面源污染治理。大力开展植树造林，绿化覆盖率达到27%以上。

　　（七）全面加强城市规划建设管理，加快城市化进程

　　城市是区域经济社会综合竞争力的重要体现，是载体、是窗口、是品牌、是形象。要在调整理顺、完善配套、积蓄力量、提高水平的基础上，加快城市规划建设管理向高品位、高质量、精细化转变。

　　城市规划要上水平。高度重视国家主体功能区规划和土地规划修编进展，努力为城市发展预留空间。城建规划要统筹考虑经贸、文化、旅游等功能需要，增加文化内涵。聘请国际国内一流专家完成对古城区、南部行政新区、徒骇河风景带及体育公园等下步发展重点区域规划的编制论证。注重阳光规划，广泛征求社会各界的意见。

　　城市建设要上水平。围绕完善江北水城总体框架，重点实施城区建设八大工程：一是完成新水河污水处理厂配套管网、徒骇河截污等环保治污工程；二是完成运河四期、铃铛湖等水系工程；三是完成利民东路、陈口路及其徒骇河大桥等交通工程；四是完成人民公园等绿化亮化工程；五是建设东聊引水管道复线工程；六是启动体育公园综合性体育馆工程；七是实施居民生活环境整治工程；八是启动龙湾小区等东昌湖经营开发工程。大力筹措城建资金，确保完成拆迁安置和城建任务，及时交付各类工程款。科学开发利用地热资源，大力发展城市公交事业，新增1万燃气用户，力争新增供热面积100万平方米。

　　城市管理要上水平。深入开展创建国家卫生城、环保模范城和园林城工作。积极稳妥地搞好“城中村”改造。进一步理顺市政管理体制机制，加强市容市貌监管，力求在城市管理、维护等方面实现新突破。切实整顿物业市场，规范物业管理。同时，各县市城区要立足各自特色和优势，切实保护好历史风貌区，规划建设好城市新区，为人民群众创造更好的工作和生活环境。

　　（八）积极扩大消费需求，进一步增强对经济社会发展的拉动力

　　消费需求是拉动经济社会发展的三大引擎之一，要高度重视扩大消费需求的拉动作用，努力增加城乡居民收入，拓展消费领域，改善消费环境，促进消费结构优化升级。

　　努力增加城乡居民收入。规范收入分配秩序，建立健全与经济发展相适应的收入增长机制。落实好工资制度改革、提高社会保障标准和离退休人员待遇等政策，引导企业建立正常的工资增长机制，在严格执行最低工资制度和小时最低工资标准的基础上，逐步增加职工工资。深入实施农民工工资支付保证金制度，严厉查处恶意拖欠工资行为。规范国有企事业单位特别是垄断行业的收入分配。

　　大力培育消费热点。引导企业开发满足不同层次消费需求的商品和服务，鼓励消费者增加文化、旅游、健身、休闲消费，提高消费水平和档次。完善住房补助政策，搞活住房二级市场，控制住房价格，促进住房消费。吸引北京、天津、济南和周边城市居民来聊城休闲消费。积极落实带薪休假制度，促进健康消费，完善健康人生，提高社会效率。努力扩大农村消费。加快推进“万村千乡”市场工程，推动中邮物流等企业开展超市、连锁店下乡进村，确保到2008年底标准化的“农家店”覆盖全市80%以上的乡镇和60%以上的村庄。

　　切实改善消费环境。加强市场监管，严厉打击假冒伪劣产品和市场欺诈行为，维护消费者权益。加强食品、药品监管，规范医疗和教育收费。支持金融机构创新金融品种，扩大消费信贷规模。加快社会保障和教育、医疗、住房等领域的改革，稳定居民支出预期，扩大即期消费。

　　（九）统筹经济社会发展，加快发展各项社会事业

　　经济发展是社会发展的基础，社会发展能更好地促进经济发展。要坚持统筹的理念，使社会事业与经济发展的阶段相适应，不断用经济发展成果惠及各项社会事业，不断改善人民群众的学习、工作、生活条件，努力促进人的全面发展，提高全社会文明素质。

　　优先发展教育事业。完善“以县为主”的义务教育管理体制，抓好农村义务教育经费保障机制改革。巩固提高“普九”水平，小学、初中在校生巩固率保持在99%和98%以上。全面完成城区小学新建扩建任务，实施农村中小学规范化建设工程。积极倡导民建公租和后勤社会化改革，鼓励民间资金投资教育事业。认真研究杜郎口中学的教改经验，进一步深化素质教育。积极发展学前教育，普及高中阶段教育，初中生升入高中阶段的比例提高6个百分点。大力发展高等教育和职业教育，为聊城大学、聊城职业技术学院创造良好办学环境，研究推进职业中专学校合并重组。积极发展成人教育、继续教育和老年教育，努力建设学习型社会。

　　加强医疗卫生服务。坚持医疗卫生的公益性质，加快建设覆盖城乡居民的基本卫生保健制度，着力缓解群众看病难、看病贵问题。新建改建200所农村社区卫生服务站、35所乡镇卫生院、83所城市社区卫生服务机构。加强公共卫生体系建设，提高疾病预防控制和突发公共卫生事件救治能力，切实做好艾滋病、乙肝、狂犬病等重大传染病的预防控制工作。加强中医工作，发挥中医药特色优势。

　　扎实做好人口与计划生育工作。贯彻优先投资于人的全面发展战略，千方百计稳定低生育水平，大力提高出生人口素质，综合治理出生人口性别比偏高问题，不断完善流动人口管理服务体系，积极应对人口老龄化。强化基层基础工作，大力开展优质服务，建立完善利益导向机制，确保人口出生率控制在13.8‰以内。

　　高度重视文化体育等各项社会事业发展。全面建设文化大市，搞好文化设施建设，大力发展城市社区文化和农村文化大院。以合唱节等大型活动为载体，广泛组织开展富有特色的群众文化娱乐活动。重视和加强各类文物和非物质文化遗产的挖掘和保护。提高竞技体育水平，开展全民健身活动。搞好第11届全运会聊城分赛场建设工作，争取国家在我市设立水上训练基地，积极承办特色体育赛事。注重国防建设，深入开展双拥共建。关心支持妇女儿童、老龄、残疾人事业，进一步做好广播电视、新闻出版、民族、宗教、对台、侨务、人防、档案、史志、气象、测绘、防震减灾等各项工作。

　　（十）切实关心群众生活，努力建设和谐社会

　　民本思想是中华民族的传统文化，也是现代文明的立足之本。要切实做到以人为本、关注民生，扎扎实实为人民群众办实事、解难事、做好事，全力维护人民群众的切身利益，努力构建和谐聊城。

　　千方百计扩大就业。切实抓好就业再就业扶持政策的落实，加强就业再就业培训和创业培训，对零就业家庭和困难群体实行就业援助。大力开发公益性岗位，重点解决“4050”下岗人员、被征地农民的再就业问题，今年新增城镇就业8万人以上。大力发展劳务经济，打造聊城劳务品牌，提高劳务输出质量，努力开拓国际劳务市场。今年新增国内劳务输出10万人、境外劳务输出1000人。积极鼓励外出务工人员回乡创业。

　　加快完善社会保障体系。完善城乡救助体系，建立被征地农民基本生活保障制度。大力发展社会福利和慈善事业，实施朝阳助学、夕阳扶老、情暖万家、康复助医、爱心助残五大救助工程。扩大社会保险覆盖面，多渠道筹措社保基金，确保基金安全和保值增值。完善企业职工基本养老保险制度，完善城镇职工基本医疗保险和失业、工伤、生育保险制度，启动实施以大病统筹为主的城镇居民医疗保险试点。大力实施安康居住工程，建设经济适用房10万平方米，为农村残疾人免费建设新居5000平方米。

　　加强平安聊城建设。完善社会治安综合治理和治安防控体系，严厉打击各类刑事犯罪。积极主动地开展矛盾纠纷排查调处工作，预防和妥善处置群体性事件。健全完善突发事件应急机制，提高有效应对突发事件的能力。认真做好人民群众来信来访工作，高度重视处理好拆迁、改制等涉及群众切身利益的问题。强化安全生产基层基础工作，建立“科技、制度、监管”三位一体的安全生产保障体系。认真做好重特大事故隐患的排查、整改和监控工作，严防各类重大恶性事故的发生。

　　加强精神文明和民主法制建设。大力弘扬社会主义核心价值体系，认真践行“八荣八耻”，发扬“自强不息、务实创新、大气开放、诚实守信”的聊城人文精神，倡导和谐理念，培育和谐精神，建设和谐文化。积极推动“诚信聊城”建设。繁荣文学艺术创作，重视发展社会科学。切实加强青少年思想道德建设，广泛开展群众性和谐创建活动。认真办好人大代表议案、建议和政协委员提案。完善基层民主管理制度，深化完善政务公开、厂务公开和村务公开制度。认真实施“五五”法制宣传教育规划，加强和改进法律援助服务。

四、加强政府管理创新与自身建设

　　全面贯彻落实胡锦涛总书记在中纪委七次全会上的重要讲话精神，全面加强各级政府工作人员思想作风、学风、工作作风、领导作风、生活作风建设，着力推进政府管理体制创新，提高行政能力和管理水平，加快建设法治政府、服务政府、责任政府和效能政府。

　　（一）切实加强学习研究。坚持解放思想、实事求是、与时俱进，认真学习新知识，研究新情况，解决新问题。全面理解、准确把握中央和省委、省政府一系列战略决策，增强贯彻落实科学发展观的自觉性和坚定性。认真学习现代经济、科技、社会管理、法律等知识，不断丰富头脑，提高驾驭全局的能力。进一步加强调查研究，认真做好结合的文章，创造性地开展工作，努力开创新局面。

　　（二）着力提高科学决策水平。抓住“充分论证、公开透明、责任追究、执行反馈”四个环节，建立健全依法科学民主决策机制。进一步健全重大事项集体决策、专家咨询、社会公示与听证、决策评估等制度。凡是涉及经济社会发展中的重大决策，必须充分听取社会各界意见；凡是与人民群众利益密切相关的重大事项，必须实行社会公示或听证；凡是做出决策的事情，必须建立健全决策反馈纠偏机制和决策责任追究制度。真正做到依法决策、科学决策、民主决策。

　　（三）大力优化发展环境。进一步转变政府职能，充分发挥市场在资源配置中的基础性作用，将主要精力放在为各类市场主体服务和创造良好发展环境上。加强对涉及人民生命财产安全领域的监管，整顿规范市场秩序。不断完善应急预案体系和社会管理制度，提高政府处置突发应急事件的能力和社会管理水平。深化行政审批制度改革，减少和规范行政审批事项，积极推行网上审批，逐步健全审批权力与审批责任挂钩机制。大力推进依法治市，全面落实依法行政“四五”规划，深化行政执法体制改革，大力推进相对集中行政处罚权工作，提高执法水平。严格控制和规范对企业的检查评比，切实治理乱收费、乱罚款、乱摊派，特别是治理公路“三乱”。全面推行行政执法责任制，加强行政复议和执法监督，完善执法评议考核、行政赔偿和过错追究制度。大力推行政务公开，不断完善政府公报、政府网站、新闻发言人、行政首长信箱和公开电话等公开渠道，保障人民群众对政府工作的知情权、参与权、表达权和监督权。

　　（四）进一步加强作风建设。坚持党的群众路线，认真解决群众关注的热点、难点问题，脚踏实地为群众办实事、解难事、做好事。大力发扬求真务实、艰苦奋斗的优良作风，努力建设节约型机关，坚决反对大吃大喝、铺张浪费。加强政府诚信建设，讲实话，办实事，言必行，行必果。坚决防止和克服官僚主义、形式主义，反对弄虚作假、虚报浮夸。切实改进会风和文风，少开会、开短会，讲短话、发短文，下决心解决文山会海问题，减少各类礼仪性、应酬性活动。要坚持真抓实干，在抓落实上狠下功夫，大力开展政府绩效评估，坚决破除本位主义，确保令行禁止、政令畅通，提高政府执行力。

　　（五）深入开展反腐倡廉工作。坚持从严治政，进一步抓好反腐倡廉工作体制机制创新，建立健全结构合理、配置科学、程序严密、制约有效的权力运行机制，确保权力运行的每一个部位、每一个环节都置于有效的监督之下。各级政府要主动接受同级人大及其常委会的工作、法律监督和政协的民主监督，高度重视新闻舆论监督和社会公众监督，支持监察、审计部门依法履行监督职责。各级政府工作人员特别是领导干部要始终牢记“两个务必”，注重培养健康的生活情趣，保持高尚的精神追求，严格遵守廉政建设的各项规定，廉洁自律，率先垂范。积极开展反腐败斗争，依法治理打击商业贿赂和职务犯罪，加大腐败案件查处力度，树立政府良好形象。

　　各位代表！我市经济社会发展已站在一个新的起点上。展望未来，我们既满怀信心，又深感责任重大。让我们更加紧密地团结在以胡锦涛同志为总书记的党中央周围，高举邓小平理论和“三个代表”重要思想伟大旗帜，认真贯彻落实科学发展观，在省委、省政府和市委的坚强领导下，同心同德，奋力开拓，以优异成绩迎接党的十七大和省、市党代会的胜利召开，在全面建设小康社会的征程上迈出新的更大的步伐！
